# Enteral resorbable diet versus standard diet in primary sphincter reconstruction: a prospective randomised trial

**DOI:** 10.1007/s00384-021-03878-x

**Published:** 2021-03-23

**Authors:** Andreas Joos, Dieter Bussen, Christian Galata, Christoph Reißfelder, Alexander Herold, Steffen Seyfried

**Affiliations:** 1 Department of Surgery, University Medical Center Mannheim, Medical Faculty Mannheim, Heidelberg University, Theodor-Kutzer-Ufer 1-3, 68167 Mannheim, Germany; 2 Deutsches End- und Dickdarm-Zentrum, Mannheim, Germany

**Keywords:** Anal fistula, fistulectomy, Primary reconstruction, Diet, Seton, Recurrence

## Abstract

**Aim:**

Bowel movements after reconstructive anorectal surgery may negatively affect surgical outcome. This study was aimed to assess any differences between a standard diet (SD) and the enteral resorbable diet (ED) in terms of operative outcomes and patient tolerance after fistulectomy with primary sphincter reconstruction.

**Method:**

Adult patients undergoing elective fistulectomy with primary sphincter reconstruction for anorectal and rectovaginal fistulas were eligible for inclusion. Patients were intraoperatively randomised to receive either the ED and peristalsis-inhibiting medication (ED) or a SD. The primary endpoint was the healing rate. Secondary endpoints included continence scores, complications and quality of life. Sample size calculation resulted in the analysis of 60 patients to detect a difference in fistula recurrence of 30% with 70% power and a 5% significance level.

**Results:**

Sixty-six patients (24 women) were prospectively and randomly assigned to the ED (*n* = 34: 51%) or a SD (*n* = 32; 48%); mean age was 47 (18-74) years. The primary healing rate was 64 out of 66 patients (96%). No statistical difference in healing rate was seen between the groups. However, patient satisfaction was significantly higher in the SD group (*P* < 0.0001).

**Conclusions:**

Fistulectomy with primary sphincter reconstruction is a safe method with low complication rates. Postoperative stool behaviour has no significant influence on the healing rate but has a significant negative impact on patient satisfaction. Therefore, maintaining a standard diet seems to be preferable following reconstructive anal surgery.

**Trial registration:**

The trial was registered with the German Clinical Trials Register (DRKS00020524).

**Supplementary Information:**

The online version contains supplementary material available at 10.1007/s00384-021-03878-x.

## Introduction

After reconstructive anorectal surgery, bowel movements can have a negative impact on surgical outcomes. Early postoperative stool passage may lead to mechanical overstretching of the suture and sphincter dehiscence; it also increases the risk of wound infection with resulting impairment of postoperative healing [1, 2].

Several different methods of preventing bowel movements are used. The most invasive is a defunctioning enterostomy. Medical bowel confinement, alone or in combination with total parenteral nutrition or enteral nutrition using absorbable nutrients, and in combination with peristalsis-inhibiting drugs, is also sometimes applied [3]. The evidence in this field is scarce.

Another trial demonstrated that enteral nutrition is preferable to intravenous nutrition for preventing defecation. The literature has not yet evaluated the question of whether any induced delay of bowel movements in the early postoperative period provides benefits after reconstructive anorectal surgery [3].

This study was aimed to assess any differences between a standard diet (SD) and the enteral resorbable diet with peristaltic inhibiting drugs (ED) in terms of operative outcomes and patient tolerance after fistulectomy with primary sphincter reconstruction.

## Method

Between April 2008 and November 2012, 66 patients were selected for the randomised trial. All subjects underwent elective fistulectomy with primary sphincter reconstruction for anorectal and rectovaginal fistulas at the Department of Colorectal Surgery of Mannheim University Hospital, Germany.

Sample size calculations indicated that 60 patients would need to be analysed to detect a difference in fistula recurrence of 30% with a 70% power and a 5% significance level. Sixty-six patients were initially chosen to allow for dropouts during follow-up.

### Patients

All consecutive patients were operated on by three colorectal surgeons. Exclusion criteria were inflammatory bowel disease, recurrent fistula, previous radiation or chemotherapy, infectious diseases (e.g. HIV) or steroid therapy as well as enterostomy. The preoperative diagnosis was confirmed by an independent specialised coloproctologist through clinical examination, proctoscopy, rectoscopy and endorectal ultrasound. Fistulas were classified as either distal fistulas, where the tract crosses the distal third of the sphincter; intermediate fistulas, where the tract crosses the middle third of the sphincter; and proximal fistulas, where the tract crosses the proximal third of the sphincter.

All patients answered questions in the two questionnaires listed below and were placed along analogue scales for pain (1 = no pain; 10 = maximum pain) and general well-being/satisfaction (1 = very satisfied; 6 = very unsatisfied).

### Questionnaires

Anorectal function was assessed using two different scores:

1. The Cleveland Clinic Incontinence Score (CCS) compares incontinence 3 months after surgery to baseline. Possible CCS scores range from 0 (continent) to 20 (totally incontinent).
2. The Rockwood Score (Faecal Incontinence Quality of Life—FIQL) evaluates different quality-of-life aspects (‘lifestyle’, 10 items; ‘coping/behaviour’, 9 items; ‘depression/self-perception’, 7 items; and ‘embarrassment’, 3 items) on a scale of 1 to 5, with 1 indicating a lower functional QoL. Patients answered the questionnaire before the operation and at three follow-up intervals (1 week, 3 months and 12 months after surgery). If direct personal contact was not possible, the survey was carried out in the form of a telephone interview.

### Randomisation

Patients were randomised prospectively by opening an envelope immediately after surgery to one of two study groups, with the surgeon and the physician responsible for follow-up visits blinded to postoperative nutritional regime. The mode of surgery was not influenced by this study.

Both groups received the same preoperative bowel lavage solution (3 l DelcoprepTM, DeltaSelect GmbH, Dreieich, Germany) and a single dose of cefazolin (2 g i.v.) or ciprofloxacin (200 mg i.v.) in patients with penicillin allergies, and metronidazole (500 mg i.v.) immediately before surgery. On the day of surgery, patients received 1000-2000 ml of isotonic crystalloid solution intravenously and 500 ml of tea or water. From the first postoperative day, both groups were allowed to drink clear liquids.

### Control group

Patients in the SD group were allowed to drink clear liquids the day of surgery and eat normal meals the day after surgery. No restrictions on eating and drinking were imposed on those patients thereafter.

### Intervention group

From the first postoperative day onwards, the RD group was offered five cups of Clinutren fruitTM (Nestlé Nutrition comp., Frankfurt, Germany) and two cups of OPD PeptamenTM (Nestlé Nutrition comp., Frankfurt, Germany). This quantity was the maximum allowed per day. In addition, patients received 2 mg loperamide once a day from the day of surgery until the 6th day after surgery. Beginning on the 7th day following surgery, this group was permitted to eat a normal diet, and no more loperamide was administered.

From the day of surgery until the 6th postoperative day, the amount of food consumed daily and the time of first defecation was documented. Pain during the first defecation was recorded on a visual analogue scale from 1 to 10 (1 = no pain; 10 = maximum pain). Patient satisfaction with the type of nutrition offered was evaluated by an analogue scale of 1 (very satisfied) to 6 (dissatisfied). Additionally, duration of hospital stay and length of time unable to work were documented. Follow-up examinations were performed after 2-4 weeks, after 6-12 weeks and every 6 months thereafter to determine or rule out fistula recurrence. The physician was not aware of whether the patient had received normal food or special enteral nutrition. In both groups, visual evaluation, digital rectal examination and proctoscopy were performed in order to detect fistula recurrence. During these follow-up examinations, continence was evaluated by Rockwood scores and CCS, and return to work was documented. The surgical technique was standardised as described before [4].

The trial was registered with the German Clinical Trials Register (DRKS00020524).

The study was approved by the local ethics committee (2017-819R-MA).

All patients gave their informed consent to participate.

## Endpoints and study visits

The main indicator used in this trial was the healing rate. This was defined as the complete healing of all tracts and external and internal openings, as well as no further secretions detected in clinical examination. All patients were evaluated after 2-4 weeks, 4-6 weeks, 6-12 weeks and every 6 months after the operation date, if possible. Wound healing after 12 weeks and beyond was defined as prolonged wound healing. Dehiscence of the adapted sphincter muscle could be easily detected 2-4 weeks after operation via inspection and palpation, as the external wound is still open at this point. The latter examination is mandatory and is performed by the operating surgeon, who is aware of the patient’s intraoperative status and the postoperative situation.

Failure to heal resulted in persisting fistulas. Primary healing with subsequent recurrence was also observed. Both outcomes were included in the term ‘recurrence’ in this trial.

Secondary endpoints were pain during the first defecation following surgery, satisfaction with the type of nutrition offered, duration of hospitalisation and length of inability to work measured as described above. The difference of the Rockwood score at 4 timepoints, CCS score before surgery and 3 months afterwards were also recorded.

## Statistical analysis

To compare the outcomes of the two groups, the following tests were applied where appropriate: Chi2 test, Fisher’s exact test, Cochran-Armitage trend test, two-sample t-test and Mann-Whitney U-test. A logistic regression analysis was not suitable due to low event rates (‘recurrence’). A test result with a *P*-value less than 0.05 is considered significant. All statistical calculations were conducted with SAS, release 9.3 (SAS Institute Inc., Cary, NC, USA).

## Results

The 66 patients in the study included 42 men and 24 women. Demographic data, as well as coloproctological history and distribution of both groups, are shown in Table 1. Primary healing rate, without any complications, after follow-up of a median 28 months (ED 26 /SD 30 month) was 89.4% (59 out of 66), or 88.2% for the ED group and 90.6% for the SD group. One patient in each group had a partial dehiscence. Both individuals were treated conservatively and did not require further operation. One patient in the RD group showed prolonged wound healing, as did 2 patients in the SD group. Both recurrences occurred in the RD group, but this difference was not significant (*P* = 0.163). After 1 year, all complications healed conservatively, resulting in a long-term healing rate of 94.1% in the RD group and 100% in the SD group. These differences were not statistically significant (Table 2).

**Table 1:** Preoperative data

		RD*	% per group	SD**	% per group	*p*
*n*		34	51.5	32	48.5	0.61
Sex	m	19	55.9	23	71.9	0.08
	w	15	44.1	9	28.1	
Age		46(18-71)		48(23-74)		0.39
Smoker	Current	7	20.6	7	21.9	0.62
	Until 12 months preoperative	3	8.8	4	12.5	
Previous proctological surgery	Any surgery	34	51.5	32	48.5	0.37
	Fistula surgery	2	5.9	6	18.8	0.08
Type of fistula	Distal	1	2.9	0	0	0.56
	Intermediate	29	85.3	31	96.9	
	High	4	11.8	1	3.1	
Length of sphincter	<3 cm	11	32.4	8	25	0.84
	3-4 cm	13	38.2	17	53.1	
	4-6 cm	10	29.4	7	21.9	
Scores preoperative	CCS median (range)	2.3 (0-8)		1.6 (0-12)		0.52
	Rockwood	3.8 (1.9-4)		3.9 (2.1-4)		0.47

*ED = enteral resorbable diet

**SD = standard diet

**Table 2:** Operative outcomes

		ED*	% (per group)	SD**	% (per group)	*p*
*n*		34	50.1	32	49.23	n.s.
Pain during first defecation	1	14	41.2	14	43.7	n.s.
	2	6	17.7	12	37.5	
	3	7	20.6	5	15.6	
	4	5	14.7	1	3.2	
	5					
	6	1	2.9			
	7					
	8	1	2.9			
	9					
Recurrence		2	5.9	0	0	n.s.
Complications	Prolonged wound healing	1		2		n.s.
	Partial dehiscence	1		1		
Hospital stay (days)	3	1	2.9	1	3.1	n.s.
	4	10	29.4	4	12.5	
	5	16	47	25	78.1	
	6	6	17.7	2	6.3	
	7	1	2.9	0		
First defecation	Day 1	10	29.4	19	59.4	*0.006
	Day 2	4	11.8	8	25	
	Day 3	1	2.9	2	6.3	
	Day 4					
	Day 5	2	5.9			
	Day 6	1	2.9			
	Not before discharge	16	47.1	3	9.4	
Days off work (days)	Mean (standard deviation)	21.4 (11.9)		26.4 (12.4)		n.s.
CCS 3 month postoperative	CCS median (range)	3.2 (0-12)		2.5 (0-12)		n.s.

*ED = enteral resorbable diet

**SD = standard diet

Participants in the SD group had a significantly earlier first postoperative defecation (*P* = 0.006). Length of hospital stay did not differ between the groups (*P* = 0.112). Most patients were discharged after 4-5 days. Overall satisfaction was significantly higher in the SD group compared with the RD group (*P* < 0.001).

There was no statistically significant difference between the SD and RD groups for pain during first defecation (*P* = 0.294) (Table 2). Time until return to work was not significantly different.

**Fig. 1:**
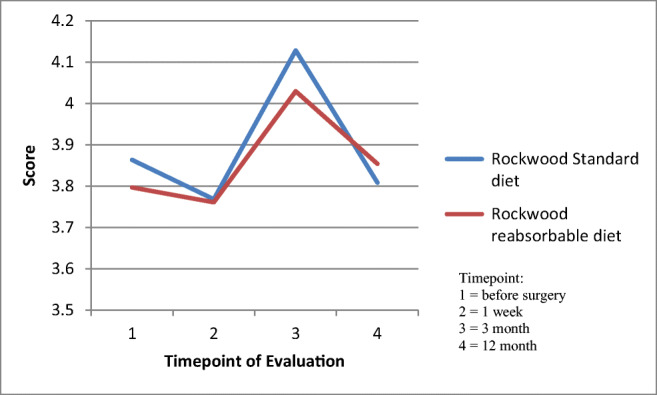
Rockwood score

The present study revealed a stable timeline in both continence scores. Values of both scores did not differ between groups (Rockwood *P* = 0.23/CCS *P* = 0.62). The course of the validated Rockwood Scores (based on the means of both groups) at the respective questionnaire time is shown in Fig. 1.

Overall, four patients (two in each group, all women) had a ventral fistula, but none had contact with the vagina.

All patients included in this study were able to complete the follow-up visits until 12 months after the operation. In some cases, the follow-ups were conducted by telephone interview at 3 and 12 months.

## Discussion

This work is the only prospectively randomised trial that examines the influence of postoperative nutrition on the outcome of fistulectomy with primary sphincter reconstruction.

**Fig. 2:**
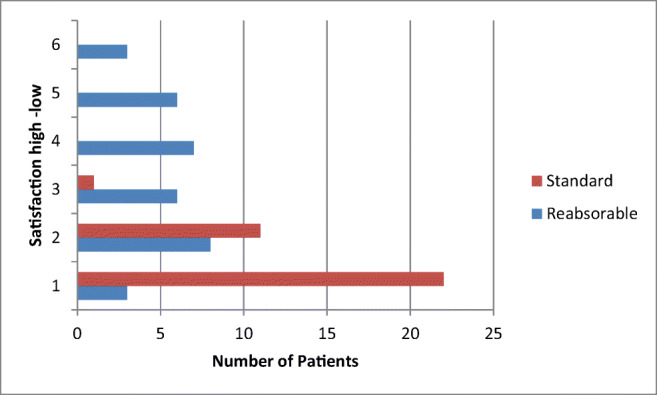
Patients satisfaction

Its main finding is that a special postoperative diet to avoid early defecation after fistulectomy with sphincter reconstruction does not improve postoperative outcomes and is therefore unnecessary. Furthermore, such a diet causes more discomfort for the patient (Fig. 2). The safety, feasibility, and excellent results of fistulectomy with primary sphincter reconstruction were again confirmed [4–8]. This trial and its results fill a gap in the existing research. Given its use of long-term follow-ups, this study’s findings on operative outcomes should also be considered valid. These results are in accordance with other (non-randomised) trials that examined the effect of nutritional regimes on the outcome of coloproctological interventions [3, 9, 10]. A retrospective British study, focused on postoperative management after sphincter reconstruction[9], divided patients into three groups: pharmacological stool prevention, protective ileostomy and normal diet with laxatives. No significant differences were found in rates of infection, wound dehiscence or surgical outcome. Constipation was more common in the pharmacological stool prevention group. An increased morbidity was observed in the protective ileostomy group.

Based on published data and this randomised trial, a special diet in the context of coloproctological surgery is discouraged. As early defecation does not influence surgical outcomes regarding sphincter reconstruction, perioperative enteral and early nutrition is advantageous. Early defecation could be avoided by a resorbable diet and inhibiting medication (loperamide). Our group has already shown that this method is feasible for avoiding defecation [3].

As the results of fistulectomy with primary sphincter reconstruction show very low complication rates and recurrences, and since the majority of these patients were operated on without oral bowel preparation, the effects of oral bowel preparation with antibiotics might be assessed as rather minimal [4]. However, studies evaluating the influence of bowel preparation specifically are lacking and might provide more conclusive evidence. On the basis of the available data, particularly recent data on oral antibiotic bowel preparation[11], no conclusive statement can be made regarding the effects of bowel preparation on operative results in coloproctology.

Since the CCS was only measured at two timepoints (preoperatively and 3 months after surgery), no graphical representation is provided. At the time this study was planned, there were no published data on fistulectomies with primary sphincter reconstruction. As later evaluations showed [4, 5, 8, 12, 13], the success rate of this method had been underestimated. In our calculations, we assumed a recurrence rate of 50% in the normal food group of 50% and 20% in the resorbable group. In retrospect, the recurrence rate was clearly overestimated, and this must be discussed as a weakness of the study.

Regardless of nutritional regime, the results show that fistulectomy with primary sphincter reconstruction is a safe therapy, since the values did not change significantly in comparing preoperative to postoperative patients. As the literature shows, continence problems after sphincter reconstructions are a rare complication.

In the only randomised study comparing fistulotomy and sphincter reconstruction with advancement flaps and postoperative incontinence[9], the results of the Wexner score (0.64 versus 0.48) were comparable. Perez et al. [14] showed that continence was improved after this reconstructive operation in patients with preoperative continence disorders, without compromising fully continent patients [4, 8, 15].

## Summary

Early postoperative defecation has no negative impact on surgical results, and certainly no impact on continence. Since the well-being of the patient is already impaired, a special diet might best be avoided. Fistulectomy with primary sphincter reconstruction is a feasible procedure resulting in a low recurrence rate that does not compromise continence. A question remains as to whether preoperative oral intestinal irrigation (especially with selective intestinal decontamination by antibiotics) is an advantage, and this should be investigated further.

## Supplementary Information

ESM 1 (DOCX 65 kb)

## Data Availability

The datasets generated during and/or analysed during the current study are available from the corresponding author on reasonable request.
