# Supplementary material for: Enteral resorbable diet versus standard diet in primary sphincter reconstruction: a prospective randomised trial
Source: Int J Colorectal Dis. 2021 Mar 23;36(7):1455–60. doi: 10.1007/s00384-021-03878-x (PMC8195750; doi:10.1007/s00384-021-03878-x)
Supplement: Supplementary file 1 — (DOCX 65 kb) [file 384_2021_3878_MOESM1_ESM.docx]

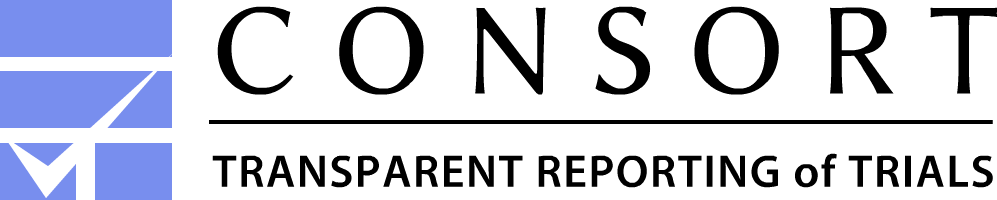


**CONSORT 2010 Flow Diagram**

## Follow-Up

Analysed (n=34)
♦ Excluded from analysis (n=0)

## Analysis

Analysed (n=32)
♦ Excluded from analysis (n=0)

Lost to follow-up (give reasons) (n= 0)

Lost to follow-up (give reasons) (n= 0)

## Enrollment

Allocated to enteral resorbable diet (n=34)

♦ Received allocated intervention (n=34)

## Allocation

Allocated to standard diet (n=32)

♦ Received allocated intervention (n= 32)

Randomized (n= 66)

Excluded (n=3)

♦  Not meeting inclusion criteria (n= 0 )

♦  Declined to participate (n= 2)

♦  Other reasons (n= 1)

Assessed for eligibility (n=69)
